# Fabriction of ZnO Nanorods with Strong UV Absorption and Different Hydrophobicity on Foamed Nickel under Different Hydrothermal Conditions

**DOI:** 10.3390/mi10030164

**Published:** 2019-02-27

**Authors:** Xin Li, Xifang Chen, Zao Yi, Zigang Zhou, Yongjian Tang, Yougen Yi

**Affiliations:** 1 Joint Laboratory for Extreme Conditions Matter Properties, Southwest University of Science and Technology, Mianyang 621010, China; lixin1010106@yeah.net (X.L.); chenxifang1988@yeah.net (X.C.); zhouzigang@swust.edu.cn (Z.Z.); 2 Sichuan Civil-Military Integration Institute, Mianyang 621010, China; tangyongjian2000@sina.com; 3 College of Physics and Electronics, Central South University, Changsha 410083, China; yougenyi@csu.edu.cn

**Keywords:** nickel foam, ZnO nanorods, hydrothermal method, ultraviolet absorption, superhydrophobic

## Abstract

ZnO nanorods have been grown on the surface of foamed nickel by a two-step method. Firstly, a layer of ZnO seed is sputtered on the surface of the foamed nickel by magnetron sputtering, and then the hydrothermal method is used to grow ZnO nanorods at different conditions (solution concentration, reaction time and reaction temperature). The results show that the morphology of ZnO nanorods is closely related to the solution concentration, reaction time, and reaction temperature. The energy band structure formed by the foamed nickel and ZnO seed layers and the growth mechanism of ZnO nanorods are discussed. The samples are characterized by Energy dispersive spectrometer (EDS), X-ray diffraction (XRD), and Raman spectroscopy. The absorption characteristics of samples to light are characterized by ultraviolet-to-visible (UV–VIS) absorption. The hydrophilicity of the samples is characterized by the static contact angle. By analyzing the performance characteristics of the samples at different conditions, we finally obtained the optimal growth parameters. At the optimal parameters, the morphology of the grown nanorods is regular, the ultraviolet band has strong absorption, and the surface of the samples forms a superhydrophobic surface.

## 1. Introduction

Foam metal refers to porous metal materials with a porosity of more than 90% and a certain strength and rigidity. Porous foam metal has been obtained from the gasification of mercury in molten aluminum by SoSnik in the United States in 1948, breaking the understanding of the dense structure of traditional metals [1]. The porous foam metal material is actually a composite material of metal and gas. It is because of this special structure that it has both metal characteristics and bubble characteristics, such as large pore size, high porosity, and low density. Therefore, porous foam metal, as a new type of functional material, has a wide range of applications in electronics, energy storage, chemical industry, and new energy [2,3,4,5,6,7,8]. At present, there are many methods for preparing foam metal: the electrochemical deposition method, sintering method, casting method, powder metallurgy method, and molten metal foaming method [9,10,11,12,13]. Nickel is a yellowish silver-white, transitional metal with magnetic properties. It is hard in air, strong in corrosion resistance, heat resistance, plasticity, and toughness, and magnetic at temperatures below 340 °C. Nickel foam is the main type of foam metal. It not only has the unique properties of nickel, but also has high porosity, large specific surface area, and unique three-dimensional network structure [14]. At present, foamed nickel is widely used in supercapacitor electrodes, catalyst carriers, fuel cells, photocatalytic, battery electrode, medicine, and other fields [15,16,17,18,19,20,21,22,23,24,25,26,27,28,29].

ZnO has a hexagonal wurtzite structure (lattice constant *a* = 0.3249 nm, *c* = 0.5206 nm), a novel wide band gap semiconductor material. The exciton binding energy of ZnO is 60 meV [30], which is much larger than 25 meV of GaN [31] and 22 meV of ZnSe [32]. As the third-generation semiconductor material, ZnO has a forbidden band width (Eg) of 3.37 eV, a high transmittance for the visible light band and a strong absorption for the ultraviolet band. In recent years, due to the unique physical properties and optoelectronic properties of low-dimensional ZnO nano-materials, it has gained wide attention, especially in the field of energy conversion of ZnO nanomaterials. Compared with ZnO bulk materials, ZnO nanomaterials have larger exciton energy because of their small size effect. Shim [33] and Gu [34] et al. grew ZnO quantum rods with a radius of a few nanometers and studied their exciton spectra. It was found that the ground state energy of excitons in ZnO quantum rods is about 0.25 eV higher than the free exciton energy of ZnO bulk materials. ZnO nanomaterials have extreme exciton energy, the electric potential of ZnO is lower than the electric potential of the dye, and its electric potential is below the lowest unoccupied energy level of the dye. When the light illuminates the dye, the photoelectrons generated by the light easily enter the conduction band of the nanomaterial, so the ZnO nanomaterial is an excellent dye-sensitized solar cell material [35]. Law [36] et al. successfully prepared ZnO nanomaterials and studied the application in dye-sensitized solar cells, and found that the solar energy conversion efficiency reached 1.5%. At the same time, based on the piezoelectric effect of ZnO, Wang [37] et al. successfully prepared the ZnO nanowire array engine, which is the world’s smallest nano-generator, and its power generation efficiency can reach about 20%. With such a generator, mechanical energy can be converted into electrical energy at the nanometer scale [38,39]. There are many methods for preparing ZnO nanomaterials, such as magnetron sputtering, hydrothermal synthesis, sol-gel, electrochemical, chemical vapor deposition [40,41,42,43,44,45,46,47,48,49,50,51,52]. Table 1 shows common methods [53,54,55,56] and information for growing ZnO nanorods. Most of the nano ZnO materials are prepared by liquid phase deposition. Among them, the hydrothermal method has mild reaction conditions and can prepare large-area samples without the generation of pollutants, so it is widely used [57].

Here, we combine the advantages of the two materials. Foam nickel as a substrate not only increases the specific surface area of ZnO nanorods, but also has the ability to absorb electromagnetic waves. In this experiment, foamed nickel was used as the substrate. First, a ZnO seed layer was deposited on the surface by magnetron sputtering. Then, the ZnO nanorods were grown by hydrothermal growth method, and the temperature, time, and reactant concentration of the reaction system were controlled, respectively. We successfully prepared 15 sets of ZnO nanorods at different conditions and analyzed the growth mechanism. We explored the surface element distribution, morphology and composition of ZnO nanorods by energy dispersive spectrometer (EDS), scanning electron microscope (SEM) and X-ray diffraction (XRD). Ultraviolet-to-visible (UV–VIS) absorption, Raman spectroscopy, and static contact angle analysis methods were used to investigate the light absorption properties, material structure and properties of ZnO nanorods. Combining all aspects of data analysis, nanorods with optimal growth parameters have regular morphology, excellent ultraviolet absorption and superhydrophobic properties.

## 2. Materials and Methods

### 2.1. Reagents and Materials

Nickel foam (porosity: 96%–97%, thickness: (1.1–2.5) ± 0.05 mm, area density: (400–500) ± 30 g/m^2^, width: 30.5 mm); deionized water; hexamethylenetetramine (HMTA); zinc nitrate hexahydrate (Zn(NO_3_)_2_∙6H_2_O); hydrochloric acid (HCl); absolute ethanol (purity 99.5%); acetone; ZnO target (purity 99.99%).

### 2.2. Cleaning of Foamed Nickel (Substrate)

The substrate was first placed in a hydrochloric acid (HCl) solution and ultrasonically cleaned for 20 min with the aim of removing inorganic ions and oxidizing substances from the surface. After washing, the substrate was ultrasonically washed twice with deionized water for 10 min each time. Then put the substrate into acetone solution and ultrasonically cleaned for 20 min, the purpose is to remove the surface oil and other organic matter. After washing, the substrate was ultrasonically washed twice with deionized water for 10 min each time. Then the substrate was placed in absolute ethanol, ultrasonically cleaned for 10 min, and then ultrasonicated with deionized water for 10 min. Finally, the foamed nickel was naturally dried.

### 2.3. Magnetron Sputtering Deposition of ZnO Seed Layer

In order to successfully grow ZnO nanorods on the surface of foamed nickel metal, we first sputtered a layer of ZnO seed on the surface by magnetron sputtering, using a purity of 99.99% and a diameter of 60 mm ZnO target. When the vacuum reached 4 × 10^−4^ Pa, the argon flow rate was adjusted to 40 sscm, and the radio-frequency (RF) sputtering power was adjusted to 60 kW. We started pre-deposition for 5 min, the purpose of which is to sputter off impurities on the surface of the target to ensure the purity of the surface deposited on the substrate during formal sputtering. In the case of formal sputtering, the substrate was aligned in the middle of the target, and the start of the glow discharge was started for 10 min. In this experiment, the deposition rate of the atom is 10 nm/min, and the thickness of the seed layer was about 100 nm after sputtering for 10 min. After the sputtering was finished, we let the machine stood still for 40 min before taking out the sample.

### 2.4. Hydrothermal Growth of ZnO Nanorods

In order to explore the conditions for hydrothermal growth of ZnO nanorods, we used the control variable method to control the solution temperature, reaction time and reaction concentration. First, the substrate on which the ZnO seed layer had been deposited was placed on a glass piece and fixed with a high temperature tape. Further, a solution of 20 mmol/L, 30 mmol/L, 40 mmol/L, 50 mmol/L, 60 mmol/L, a ratio of 1:1, and a volume of 80 mL was prepared using hexamethylenetetramine (HMTA) and zinc nitrate hexahydrate (Zn(NO_3_)_2_·6H_2_O). For the first set of experiments, we controlled the solution concentration and placed the sample in five different concentrations of solution with a reaction time of 4 h and a reaction temperature of 95 °C. For the second set of experiments, we controlled the reaction time of 2, 3, 4, 5, and 6 h with the reaction concentration set to 40 mmol/L and a reaction temperature of 95 °C. For the third group of experiments, we controlled the reaction temperature respectively 75 °C; 85 °C; 95 °C; 105 °C; 115 °C with the reaction time was 4 h and the reaction concentration was 40 mmol/L. Finally, the sample was washed several times with deionized water and then dried at room temperature.

### 2.5. Characterization

Scanning electron microscopy (SEM), energy dispersive spectroscopy (EDS) and X-ray diffraction (XRD) were used to analyze the morphology, elemental composition, and structural composition of the samples. The samples were passed through a Raman spectrometer (Renishaw In via) with a laser wavelength of 514.5 nm, a spectral resolution of 1–2 cm^−1^, and a spot diameter of 1–2 um to obtain a Raman spectrum. The sample was passed through a solid UV–VIS–NIR spectrophotometer (UV-3700, Shimadzu, Kyoto, Japan) with a resolution of 0.1 nm, and the wavelength range was 185–3300 nm to obtain an optical absorption diagram. The static contact angle test uses a contact angle tester (DSA30, Krüss, Hambourg, Germany) with a contact angle range of 0–180° and a resolution of ±0.01°.

## 3. Results and Discussion

In order to grow complete nanorods on the surface of the foamed nickel, we first sputtered a layer of ZnO seed with magnetron, and then the nanorods in the solution start to grow with the seed substrate as the nucleus. The reaction of the solution system is as follows:

(CH_2_)_6_N_4_ + 6H_2_O → 6HCHO + 4NH_3_ (1–1)

NH_3_∙H_2_O ⇌ NH_4_^+^ + OH^−^ (1–2)

Zn^2+^ + 2OH^−^ + 2H_2_O = Zn(OH)_4_^2−^ (growth unit) + 2H^+^ (1–3)

Zn(OH)_2_ → ZnO + H_2_O (1–4)

In the reaction system, hexamethylenetetramine (HMTA) is first hydrolyzed to form NH_3_, and then NH3 is hydrolyzed again to form OH^−^, and OH^−^ reacts with Zn^2+^ in zinc nitrate to form Zn(OH)_2_. Zn(OH)_2_ colloid first synthesized Zn(OH)_4_^2−^, and then formed crystal nucleus with certain structure through oxygen bridge cooperation between growing elements and the proton reaction of anionic group. Next, the growth unit is superposed on the crystal interface by dehydration reaction and grows into a crystal structure. The growth rate of the crystal is related to the supersaturation of the solution, and the lower the supersaturation, the lower the growth rate of the crystal. As the reaction proceeds, the reactants are consumed, the supersaturation is reduced, and the growth rate difference in all directions is increased. In other words, the growth state of each crystal orientation is different.

**Figure 1:**
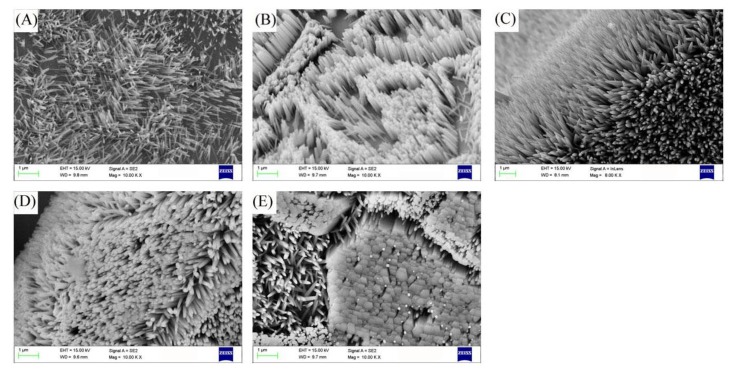
Scanning electron microscopy (SEM) image of ZnO at different solution concentrations: (**A**) 20 mmol/L, (**B**) 30 mmol/L, (**C**) 40 mmol/L, (**D**) 50 mmol/L, and (**E**) 60 mmol/L.

In Figure 1A, we can observe that at the solution concentration of 20 mmol/L, the small precursor concentration and small driving force lead to slow growth rate of crystal seeds and low mass fraction of crystal seeds, forming sparse nanorods and short rod-like nanorods. Figure 1B shows that at a solution concentration of 30 mmol/L, the increase in driving force leads to a significant increase in the number of ZnO nanorods. At this time, the diameter of the nanorods are about 300 nm, hexagonal structure is obvious. In Figure 1C, when the solution concentration is 40 mmol/L, the crystal growth rate is fast, and a large area of ZnO nanorods have been grown. The nanorods shape are regular, and the hexagonal diameter becomes smaller at about 100 nm. Figure 1D shows that the nanorods structure are dense when the solution concentration is 50 mmol/L. The hexagonal diameter of the nanorods increases, and its diameter is about 200 nm. Figure 1E shows that when the solution concentration is 60 mmol/L, the nanorods are hexagonal and the diameter of about 500 nm. The nanorods are tightly bonded together and are gradually growing in the direction of forming a ZnO solid structure.

**Figure 2:**
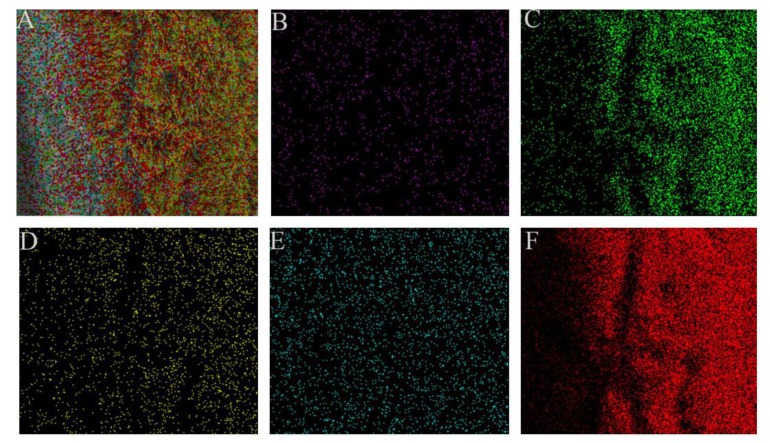
Element distribution at Solution concentration 40 mmol/L, reaction time 4 h, reaction temperature 95 °C: (**A**) Overall distribution of elements. (**B**) Purple: C element. (**C**) Green: O element. (**D**) Yellow: Al element. (**E**) Blue: Ni element. (**F**) Red: Zn element.

When the electron beam interacts with the nickel foam, the X-ray characteristic wavelength of each element on the surface of the nickel foam is excited and finally detected by the detector. Based on the detected characteristic wavelength, we can know the element type and distribution of the substance. Figure 2A shows the elemental species and distribution of the surface of the foamed nickel. Figure 2B shows that the C element is contained therein because the presence of organic substances, such as grease in the air, is easily adsorbed by the surface of the substance. Figure 2C,F show that the distribution of oxygen and zinc is the most dense and extensive. Figure 2D shows that the element of Al is contained because the Al sample stage is used. When the spectrum is scanned in a relatively thin area, there will be a signal from the sample stage. In Figure 2E, from the distribution of nickel, we can see the porous shape of foamed nickel.

**Figure 3:**
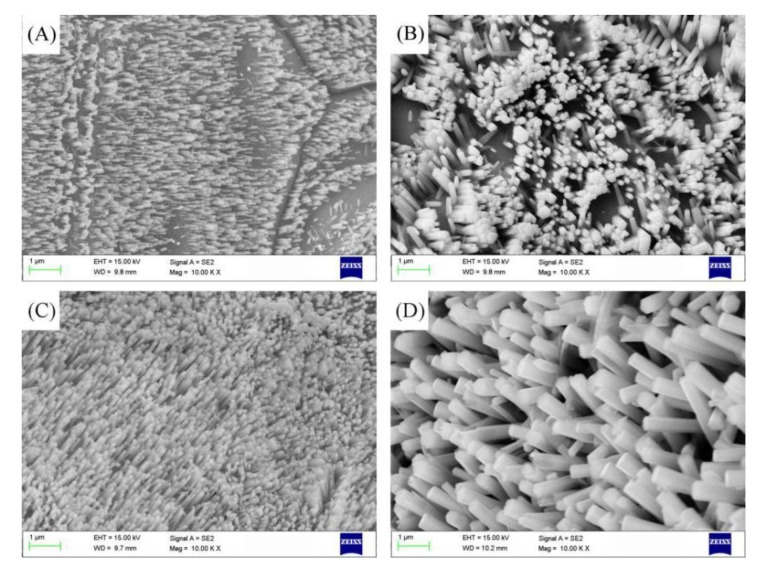
SEM image of ZnO at different reaction times: (**A**) 2 h, (**B**) 3 h, (**C**) 5 h, and (**D**) 6 h.

Figure 3A,B shows that the reaction time is short and the nanorods are sparse and short. The nanorods have an increasing trend in both axial and radial directions. Figure 3C,D shows that the reaction time is long and the structure is dense. Nanorods appear to be interconnected and top dissolution.

**Figure 4:**
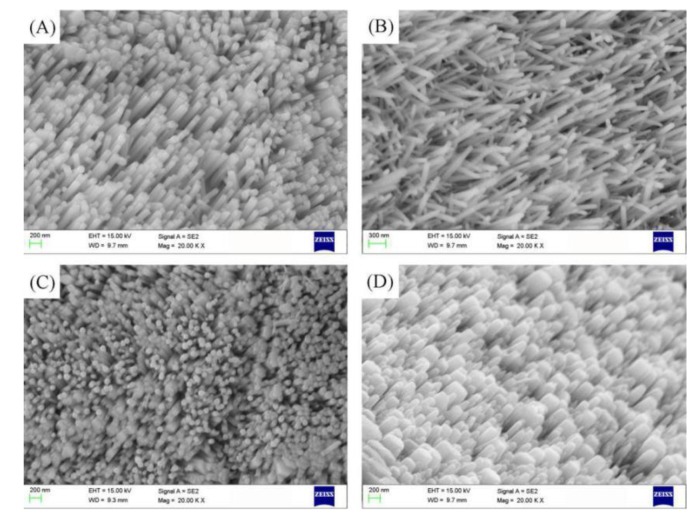
SEM image of ZnO at different reaction temperatures: (**A**) 75 °C, (**B**) 95 °C, (**C**) 105 °C, and (**D**) 115 °C.

The formation of nanorods is an endothermic reaction, and at the low temperature, reaction will be relatively slow. In Figure 4A,B, it can be seen that the reaction temperature is low and the surface obtain nanorods with a large area, but the growth of the nanorods are incomplete. In this case, when the concentration and time are certain, the temperature mainly affects the forming quality of the nanorods. Figure 4C,D show that the high reaction temperature leads to overgrowth of the nanorods and the formation of ZnO nanorods are dense.

In general, the growth rate of the nanorods in the axial direction is greater than the radial growth rate. When the concentration of the precursor solution is too low, the driving force is insufficient. Therefore, nanorods grow slowly, and it takes a longer period of time to grow intact nanorods. The concentration of the precursor solution is too high, the driving force is large, the nanorod growth rate is fast, and the growing nanorods and other surrounding nanorods are closely arranged to grow toward the shape of the solid block. The reaction time is short, and the nanorod growth is incomplete. When the reaction time is too long, the nanorod overgrowth leads to deformation. The temperature mainly affects the speed of particle migration in solution and further affects the forming quality of nanorods. After the particles are deposited to form crystal nuclei, the growth of the nanorods begins and the diameter increases. As the reaction time, temperature, and concentration increase, then the diameter of the nanorods becomes smaller. When the nanorods grow completely, the nanorods can no longer continue to grow without limit. Since the reaction system is alkaline, ZnO is an amphoteric oxide. When the nanorods grow to a certain extent, the deposition rate of ZnO is less than the dissolution rate, and the top of the ZnO nanorods will dissolve. At the same time, the nanorods are closely arranged to adhere to each other, and then the bulk nanorods are formed. Overall, at solution concentration of 40 mmo/L, reaction time of 4 h and reaction temperature of 95 °C, the nanorods are arranged in a regular manner (divergent arrangement) and have the best growth morphology.

**Figure 5:**
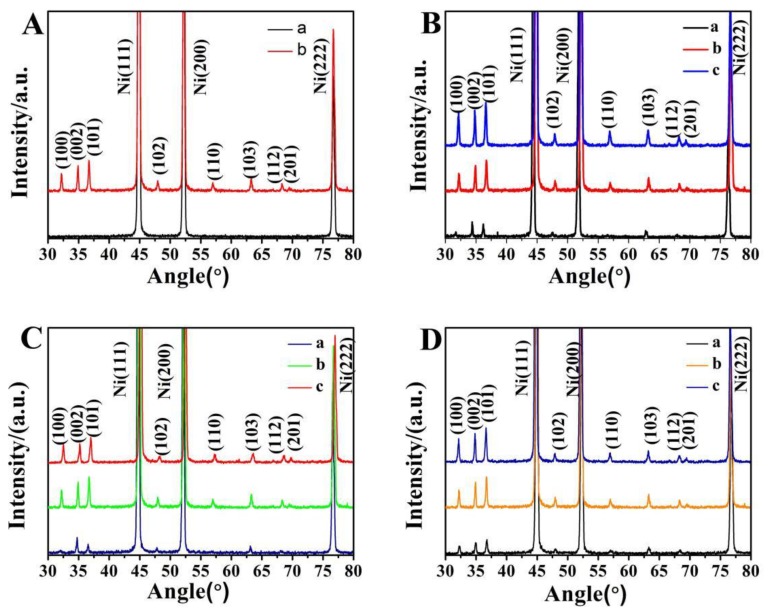
(**A**) (a): XRD images of well-grown nanorods, and (b): XRD images of ungrown nanorods. (**B**) XRD images of ZnO grown at different concentrations: (a) 20 mmol/L, (b) 40 mmol/L, and (c) 60 mmol/L. (**C**) XRD images of ZnO grown at different times: (a) 2 h, (b) 4 h, and (c) 6 h. (**D**) XRD images of ZnO grown at different temperatures: (a) 75 °C, (b) 95 °C, and (c) 115 °C.

Figure 5A shows that the diffraction peaks of foamed nickel correspond to (111), (200), and (222) crystal orientations at 44.5°, 51.9°, and 76.7°, respectively. The nanorods correspond to the wurtzite ZnO structure, and no diffraction peaks other than ZnO are found. The crystal orientation of the nanorods includes (100), (002), (101), (102), (110), (103), (112), (201). Due to the three-dimensional network structure of foamed nickel, the diffraction peak of the (101) crystal orientation is strong. Figure 5B,C shows that the reaction time is short and the solution concentration is low, so the peak of (100) crystal orientation is not obvious, and the growth of (002) crystal orientation is dominant. In Figure 5D, the main peaks of diffraction are expressed at different temperatures. As the temperature increases, the intensity of the main peak of the diffraction also increases, but the range of variation is not large. At solution concentration of 40 mmol/L, reaction time of 4 h and reaction temperature of 95 °C, not only the intensity of each crystal diffraction peak is strong, but also the line width is narrow, which indicates that the nanorods have high crystallinity.

**Figure 6:**
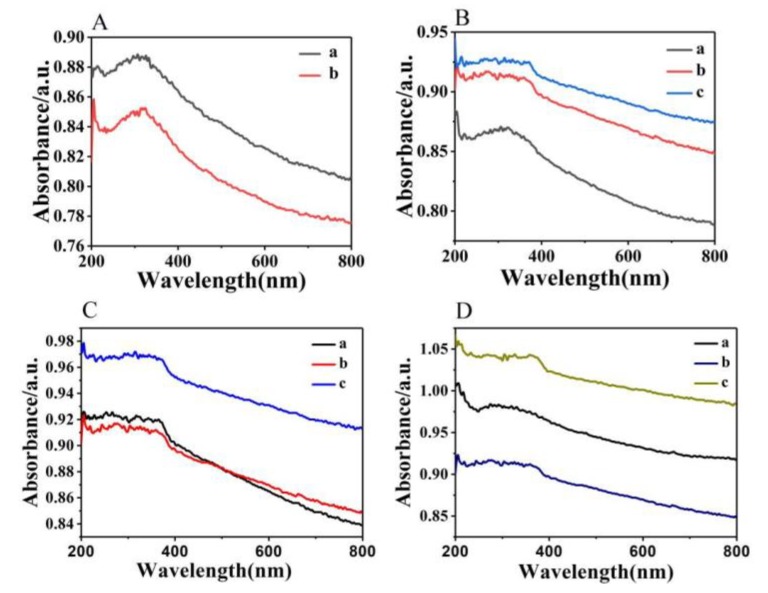
(**A**) (a): Ultraviolet-to-visible (UV–VIS) absorption foamed nickel in growing seed layers, and (b): UV–VIS absorption of foamed nickel. (**B**) UV–VIS absorption of ZnO grown at different solution concentrations: (a) 20 mmol/L, (b) 40 mmol/L, and (c) 60 mmol/L. (**C**) UV–VIS absorption of ZnO grown at different reaction times: (a) 2 h, (b) 4 h, and (c) 6 h. (**D**) UV–VUS absorption of ZnO grown at different reaction temperatures: (a) 75 °C, (b) 95 °C, and (c) 115 °C.

Figure 6A shows that nickel foam has a strong absorption of light in the ultraviolet range, but there is obvious fluctuation in light absorption in the 200–400 nm optical band. After sputtering a layer of ZnO seed on the surface of the foamed nickel, the light absorption peak is broadened in the 200–400 nm optical band; due to the scattering effect of light, the phenomenon of spectrum offset (blue shift) is exhibited. Figure 6B shows that when the solution concentration is low, the absorption of light mainly comes from foamed nickel, due to the growth of ZnO nanorods are sparse and short. Since the nanorods on the surface of the foamed nickel are closely arranged in a large area, the absorption of light is the strongest at higher solution concentration. As shown in Figure 6C, when the reaction time is 4 h, the diameter of the ZnO nanorods are small and the length to diameter ratio is the largest. Therefore, when light illuminates the surface of the material, the ability to capture light is relatively weak. Compared with the reaction time of 4 h and the reaction time of 2 h, the number of nanorods grown in 4 h is large, but the diameter of the nanorods is small; the number of nanorods grown in 2 h is small, but the diameter is large. Therefore, in the wavelength of 400–800 nm, the light absorption abilities of the two are substantially equal. When the reaction time is long, the diameter of the nanorods is large. The nanorods on the surface of the substrate are connected together in a large area, so the absorption of light is strongest. Figure 6D shows that, at the temperature of 75 °C, the absorption peak decreased at the wavelength of around 250 nm due to incomplete growth of the nanorods. According to the SEM image under different temperature conditions in Figure 4, large-area nanorods can be grown on the surface of the substrate at different temperatures. Since the diameter of the nanorods are different at different temperatures, the light absorption capacity is different. The larger the diameter of the nanorods, the stronger the ability to capture light.

From the Figure 6, we can see that the nanorods have strong absorption in the ultraviolet band (wavelength 200–400 nm). Due to the small size of the nanorods, the arrangement of atoms around the nanorods does not have long-range order and, therefore, no longer has the properties of solid ZnO. This phenomenon is the same as two-dimensional metal nanomaterials [58]. ZnO nanorods have a quantum size effect, which causes the energy level to separate into energy bands and the band gap increases. Thus, the absorption at short wavelengths is strong, thus exhibiting broadband absorption in the ultraviolet band. Due to this light absorption property, it may be used in the field of ultraviolet detectors [59], photocatalysis [23,24,25], etc.

**Figure 7:**
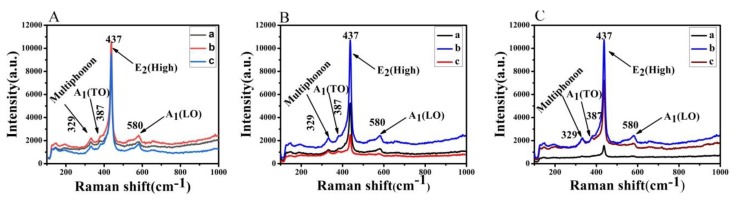
(**A**) Raman spectroscopy of ZnO grown at different solution concentrations: (a) 20 mmol/L, (b) 40 mmol/L, and (c) 60 mmol/L. (**B**) Raman spectroscopy of ZnO grown at different reaction times: (a) 2 h, (b) 4 h, and (c) 6 h. (**C**) Raman spectroscopy of ZnO grown at different reaction temperatures: (a) 75 °C, (b) 95 °C, and (c) 115 °C.

ZnO nanorods belong to polar nanocrystalline semiconductors and have the characteristics of amorphous spectrum. The four peaks appearing in Figure 7 are 329 cm^−1^, 387 cm^−1^, 437 cm^−1^, 580 cm^−1^. Among them, the 387 cm^−1^, 437 cm^−1^, 580 cm^−1^ mode is a first-order scattering mode, and the 329 cm^−1^ mode is a second-order scattering mode. The strongest Raman peak of 437 cm^−1^ comes from the optical phonon high-frequency vibration mode E_2_, which is a typical characteristic peak of wurtzite ZnO [53]. The 329 cm^−1^ peak belongs to the effect of multiphonon scattering superposition. The 387 cm^−1^ peak belongs to the transverse optical mode. The peak of 580 cm^−1^ is related to defects such as oxygen vacancies, zinc gaps and their complexes [60,61]. In the Figure 7, when the temperature is low and the reaction time is long, the diffraction peak of the nanorod is not obvious. When the solution concentration of the solution is different, the three sets of diffraction peaks of the Raman spectrum are not much different. At the concentration of 40 mmol/L, the reaction temperature of 95 °C and the reaction time of 4 h, the intensity of scattered light of the nanorods is the largest, indicating that the nanorods have high crystallinity and quality. Through Raman spectroscopy, we know that its diffraction intensity is strong, so the absorption of light is not the strongest.

**Figure 8:**
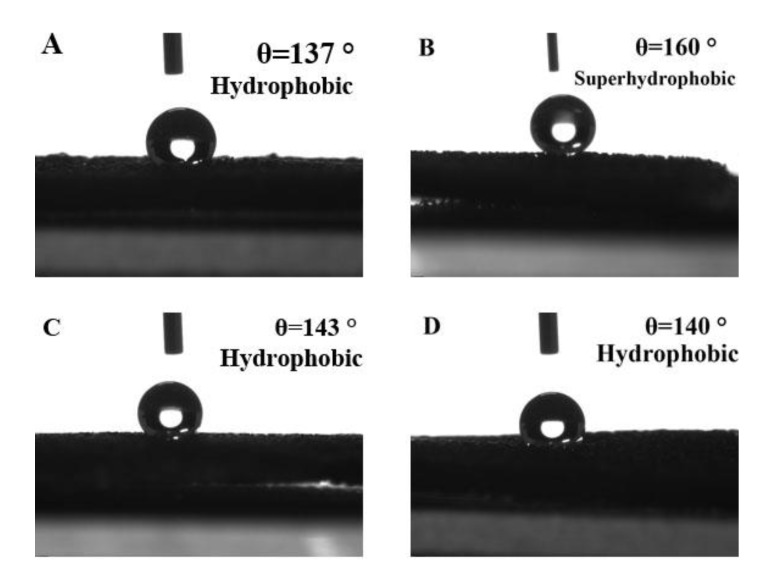
Static contact angle at different conditions: (**A**) solution concentration: 20 mmol/L, reaction temperature: 95 °C, reaction time: 4 h; (**B**) solution concentration: 40 mmol/L, reaction temperature: 95 °C, reaction time: 4 h; (**C**) solution concentration: 40 mmol/L, reaction temperature: 75 °C, reaction time: 4 h; and (**D**) solution concentration: 40 mmol/L, reaction temperature: 115 °C, reaction time: 4 h.

ZnO nanorods grown on the surface of foamed nickel can greatly change the hydrophobicity of the surface of the material. According to Young’s formula, If the contact angle θ < 90°, the surface of the material is wet or partially wet; if the contact angle θ > 90°, the surface of the material is not wet; if the contact angle θ > 150°, the surface of the material may be referred to as a superhydrophobic surface. When the number of nanorods on the surface of the foamed nickel surface is small, the contact angle is relatively small as shown in Figure 8A. When the number of nanorods on the surface of the foamed nickel is large, nanorods have small diameters and exhibit divergence arrangement, so the contact angle is the largest as shown in Figure 8B; the other is that when the diameter of the nanorod is large, the flatness is higher and the contact angle is smaller as shown in Figure 8C,D. Figure 8B shows the formation of a superhydrophobic surface on the surface of the material, which is expected to improve the application of nanomaterials in biological tissue engineering [62]. The angle between the Raman spectral line and the horizontal line (abscissa) in Figure 7 can indicate the uniformity of the surface of the material. The larger the angle, the more irregular the surface of the material. The results of Figure 8 and Figure 7 correspond to each other.

## 4. Conclusions

In summary, the foamed nickel substrate provides a special three-dimensional network structure that allows the nanorods on the surface to have a large specific surface area. Second, magnetron sputtering reduces the probability of lattice mismatch between ZnO nanorods and foamed nickel. Finally, compared with other methods, the hydrothermal method for preparing ZnO nanorods is more efficient, non-polluting, and simple to operate. The solution concentration, reaction time, and reaction temperature factors are different for the growth of ZnO nanorods. Combining all aspects of performance, we obtained the best growth parameters: solution concentration of 40 mmol/L, reaction time of 4 h, and reaction temperature of 95 °C. The nanorods at the optimal parameters, because of the small diameter of the nanorods, are not the strongest absorption of light, but also exhibit strong absorption of light. It can be seen from the SEM diagram that the nanorods with the best parameters have best growth morphology. Raman spectroscopy shows that the best parameters have the strongest diffraction peak and the nanorods have high quality. The static contact angle shows that the nanorods grown at the optimal parameters make the surface of the material a superhydrophobic surface, so it can be used as a superhydrophobic material. The growth of ZnO nanorods on the surface of foamed nickel is expected to achieve new changes in the field of photoelectric conversion due to its strong light absorption capacity and strong hydrophobic properties.

## Figures and Tables

**Table 1:** Average diameter and average length of ZnO nanorods grown by common methods.

Growth Method	Avg Nanorod Length (nm)	Avg Nanorod Diameter (nm)
Hydrothermal synthesis	2000	150
Organometallic method	25	3
Wet chemical method	2000	250
Via sonochemical method	6000	150
